# JNK suppression of chemotherapeutic agents-induced ROS confers chemoresistance on pancreatic cancer stem cells

**DOI:** 10.18632/oncotarget.2693

**Published:** 2014-11-19

**Authors:** Shuhei Suzuki, Masashi Okada, Keita Shibuya, Manabu Seino, Atsushi Sato, Hiroyuki Takeda, Shizuka Seino, Takashi Yoshioka, Chifumi Kitanaka

**Affiliations:** ^1^ Department of Molecular Cancer Science, Yamagata University School of Medicine, Yamagata 990-9585, Japan; ^2^ Department of Clinical Oncology, Yamagata University School of Medicine, Yamagata 990-9585, Japan; ^3^ Department of Regional Cancer Network, Yamagata University School of Medicine, Yamagata 990-9585, Japan; ^4^ Oncology Research Center, Research Institute for Advanced Molecular Epidemiology, Yamagata University, Yamagata 990-9585, Japan; ^5^ Global COE program for Medical Sciences, Japan Society for Promotion of Science, Tokyo 102-8471, Japan; ^6^ Department of Obstetrics and Gynecology, Yamagata University School of Medicine, Yamagata 990-9585, Japan; ^7^ Department of Neurosurgery, Yamagata University School of Medicine, Yamagata 990-9585, Japan; ^8^ Research Institute for Promotion of Medical Sciences, Yamagata University School of Medicine, Yamagata 990-9585, Japan

**Keywords:** cancer initiating cells, chemotherapy, combination therapy, c-Jun N-terminal kinase

## Abstract

Chemoresistance associated with cancer stem cells (CSCs), which is now being held responsible for the pervasive therapy resistance of pancreatic cancer, poses a major challenge to the successful management of this devastating malignancy. However, the molecular mechanism underlying the marked chemoresistance of pancreatic CSCs remains largely unknown. Here we show that JNK, which is upregulated in pancreatic CSCs and contributes to their maintenance, is critically involved in the resistance of pancreatic CSCs to 5-fluorouracil (5-FU) and gemcitabine (GEM). We found that JNK inhibition effectively sensitizes otherwise chemoresistant pancreatic CSCs to 5-FU and GEM. Significantly, JNK inhibition promoted 5-FU- and GEM-induced increase in intracellular reactive oxygen species (ROS), and scavenging intracellular ROS by use of N-acetylcysteine impaired JNK inhibition-mediated promotion of the cytotoxicity of 5-FU and GEM. Our findings thus suggest that JNK may contribute to the chemoresistance of pancreatic CSCs through prevention of chemotherapeutic agents-induced increase in intracellular ROS. Our findings also suggest that JNK inhibition combined with 5-FU- and/or GEM-based regimens may be a rational therapeutic approach to effectively eliminate pancreatic CSCs.

## INTRODUCTION

Pancreatic cancer is among the most intractable of all human cancers and is also a leading cause of cancer-related death in the world [1, 2]. Besides the difficulty of early detection, which leads to dismally low surgical resection rate, pancreatic cancer is characterized by pervasive therapy resistance, being refractory to both chemotherapy and radiotherapy [3–5]. In the past decades, the survival of patients with pancreatic cancer has been improved with the development of fluorouracil (FU)- and gemcitabine (GEM)-based chemotherapeutic regimens, however, the prognosis of pancreatic cancer still remains miserably poor with only less than 10% of patients surviving 5 years [5–7].

Cancer stem cells (CSCs), a subpopulation of cancer cells specifically endowed with tumor-initiating capacity, have often been associated with higher therapy resistance compared with non-stem cancer cells and are therefore increasingly deemed a major culprit of post-treatment progression and/or recurrence of a variety of, albeit not all, human cancers [8–12]. Since the identification of pancreatic CSCs, studies have consistently shown that pancreatic CSCs do have higher chemoresistance than non-stem pancreatic cancer cells and have also implicated pancreatic CSCs in the poor prognosis of pancreatic cancer, suggesting that overcoming the chemoresistance inherently associated with pancreatic CSCs is apparently an indispensible step to improve overall management of pancreatic cancer [13, 14]. However, to date, the molecular basis on which pancreatic CSCs acquire resistance to conventional chemotherapeutic agents against pancreatic cancer has been poorly understood.

Here in this study, while investigating the molecular mechanism underlying the differential chemoresistance to 5-FU and GEM between pancreatic CSCs and their non-stem counterparts, we discovered that the JNK pathway is critically involved in pancreatic CSCs' resistance to these chemotherapeutic agents through suppression of the intracellular level of reactive oxygen species (ROS). Our findings suggest that targeting this JNK – “ROS defense” axis in combination with current chemotherapeutic regimens may be a rational approach to overcome the therapy resistance of pancreatic cancer.

## RESULTS

### Self-renewing pancreatic cancer stem cells are highly resistant to 5-fluorouracil and gemcitabine compared with their differentiated counterparts

**Figure 1:**
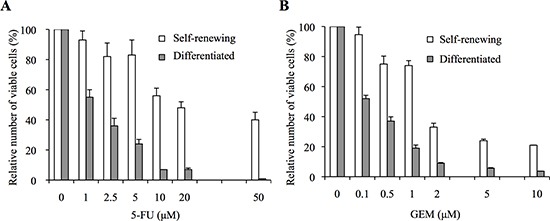
Pancreatic cancer stem cells show higher resistance to 5-fluorouracil and gemcitabine than their non-stem cell counterparts **(A, B)** PANC-1 CSLCs, either maintained under the stem cell culture condition (Self-renewing, white bars) or induced to undergo differentiation in the presence of serum (Differentiated, gray bars), were treated with the indicated concentrations of 5-fluorouracil (A, 5-FU) or gemcitabine (B, GEM) for 3 days. Then, the number of viable cells was determined using trypan blue as a vital dye. Values in the graphs are expressed as relative to controls (i.e., drug concentration = 0 μM) and represent means + SD from triplicate samples of a representative experiment repeated with similar results.

CSCs are in general considered to be highly resistant to chemotherapeutic drugs [9, 10]. To examine whether this applies to the pancreatic CSCs we have established, we used PANC-1 CSLCs, CSCs derived from the pancreatic cancer cell line PANC-1 [15], and determined the sensitivity of self-renewing (undifferentiated) PANC-1 CSLCs and their serum-differentiated counterparts to 5-FU and GEM, which are current chemotherapeutic agents of choice for the treatment of pancreatic cancer. Treatment of self-renewing and differentiated PANC-1 CSLCs with 5-FU for 3 days reduced the number of viable cells in a concentration-dependent manner, with the IC_50_ values being 16.8 μM and 1.7 μM, respectively (Figure 1A). Essentially similar results were obtained when GEM was used instead of 5-FU, with the IC_50_ values being 1.5 μM and 0.12 μM for self-renewing and differentiated PANC-1 CSLCs, respectively (Figure 1B). Thus, self-renewing PANC-1 CSLCs were by far more resistant to 5-FU and GEM than their differentiated counterparts. We then conducted similar analyses using PSN-1 CSLCs, CSCs derived from another pancreatic cancer cell line PSN-1 [15]. The results indicated that the IC_50_ values of 5-FU and GEM for self-renewing PSN-1 CSLCs were again much higher than those for their differentiated counterparts [5-FU: approximately 1 μM and 0.3 μM for self-renewing and differentiated PSN-1 CSLCs, respectively; GEM: 0.11 μM and < 0.01 μM for self-renewing and differentiated PSN-1 CSLCs, respectively] (Supplementary Figure S1). Thus, the self-renewing stem cell status was indeed associated with a highly chemoresistant phenotype in pancreatic cancer cells in this study.

### Pivotal role of JNK in the maintenance of the chemoresistant phenotype of pancreatic cancer stem cells

**Figure 2:**
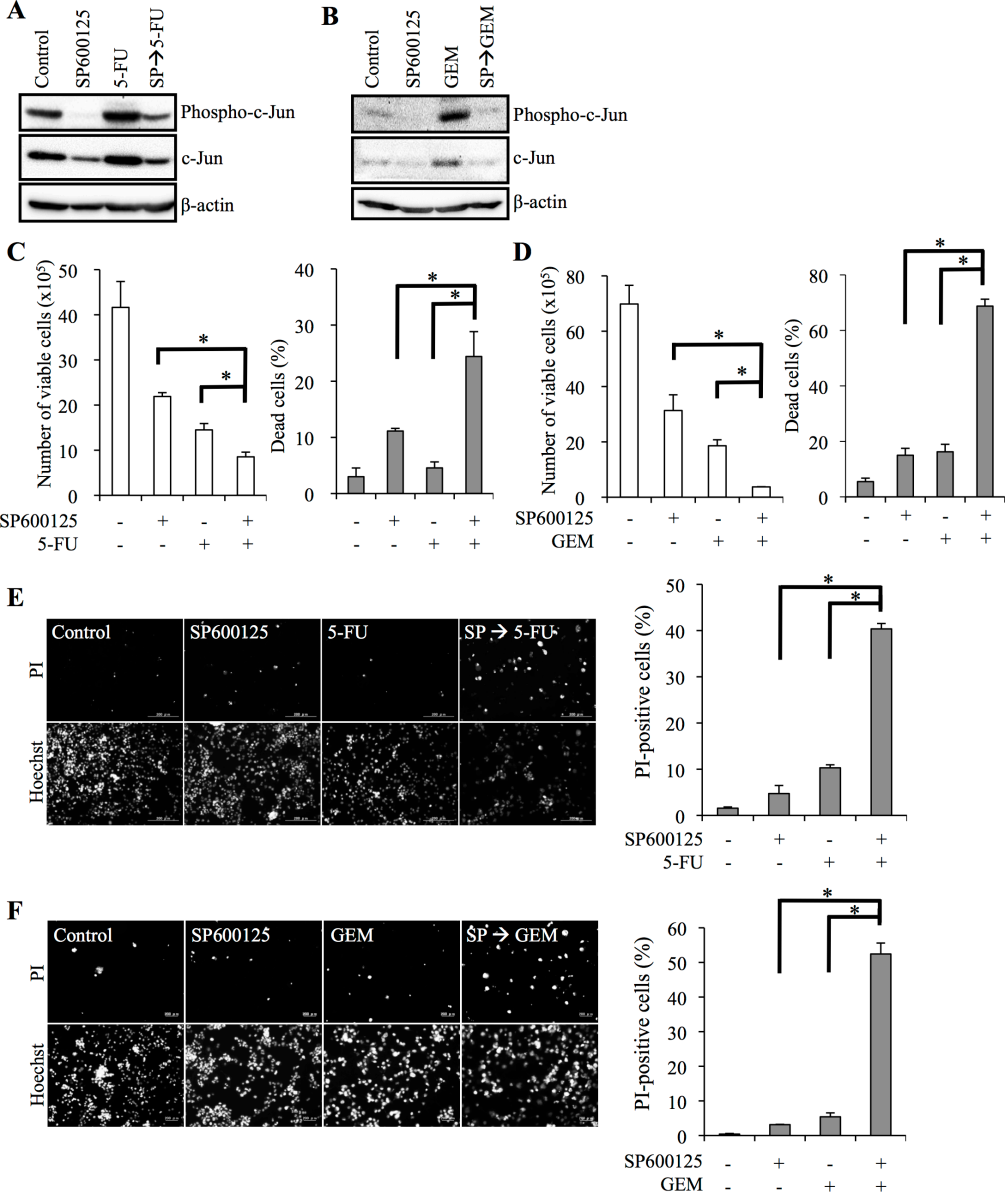
JNK inhibitor pretreatment sensitizes pancreatic cancer stem cells to 5-fluorouracil and gemcitabine **(A-F)** PANC-1 CSLCs pretreated with or without SP600125 (SP, 20 μM) for 3 days and subsequently treated with or without 5-fluorouracil (5-FU, 20 μM) or gemcitabine (GEM, 2 μM) as indicated for 3 days in the absence of SP600125 were analyzed as follows. **(A, B)** Cells were subjected to immunoblot analyses for the expression of phospho- and total c-Jun. **(C, D)** The number of viable cells (*left panels*) and the percentage of dead cells (*right panels*) were determined using trypan blue as a vital dye. Values represent means + SD from triplicate samples of a representative experiment repeated with similar results. *P < 0.05. **(E, F)** Cells were subjected to cell death analysis using propidium iodide (PI) as a vital dye. *Left,* representative fluorescence images of PI-(*upper rows*) and Hoechst-(*lower rows*) positive cells are shown. *Right*, the percentage of PI-positive cells (dead cells) relative to Hoechst-positive cells (total cells) was determined. Values in the graphs represent means + SD from triplicate samples of a representative experiment repeated with similar results. *P < 0.05.

We have recently demonstrated that the JNK signaling pathway is activated in self-renewing pancreatic CSCs compared with their differentiated counterparts [15]. Because our present data indicated that self-renewing pancreatic CSCs were more resistant to 5-FU and GEM (Figure 1; Supplementary Figure 1), we questioned whether JNK signaling is involved in the chemoresistance of pancreatic CSCs to these drugs. To address this question, we determined whether the effects of 5-FU/GEM on pancreatic CSCs were altered by treatment with the JNK inhibitor SP600125, which effectively inhibited JNK activity in both PANC-1 CSLCs and PSN-1 CSLCs (Figure 2A, 2B; Supplementary Figure S2A, S2B). When the pancreatic CSCs were exposed to 5-FU or GEM for 3 days after first treating them with SP600125, the number of viable cells decreased and the proportion of dead cells increased significantly compared with 5-FU or GEM treatment alone (Figure 2C, 2D; Supplementary Figure S2C, S2D), suggesting that SP600125 sensitized pancreatic CSCs to these drugs through promotion of 5-FU/GEM-induced cell death. For confirmation, cell death was also analyzed by treating cell cultures in situ with a fluorescent vital dye propidium iodide (PI) instead of using trypan blue, which enabled us to count the number of dead cells without losing dead cells during the cell harvesting procedure. The results were essentially similar to those obtained by using trypan blue as a vital dye, with SP600125 pretreatment combined with 5-FU/GEM treatment causing remarkable cell death in pancreatic CSCs. In contrast, treatment with 5-FU/GEM alone again had little effect on cytotoxicity (Figure 2E, 2F; Supplementary Figure S2E, S2F).

We also asked here whether the timing of treatment affects the effect of SP600125. PANC-1 CSLCs were either treated with SP600125 before 5-FU treatment (pre-treatment), treated simultaneously with SP600125 and 5-FU (co-treatment), or treated with SP600125 before and simultaneously with 5-FU (pre- and co-treatment). The results indicated that “co-treatment” was significantly less effective than “pre-treatment” and “pre- and co-treatment” (Supplementary Figure S3), suggesting that inhibition of JNK prior to 5-FU treatment may be critical for efficient sensitization of pancreatic CSCs to 5-FU.

**Figure 3:**
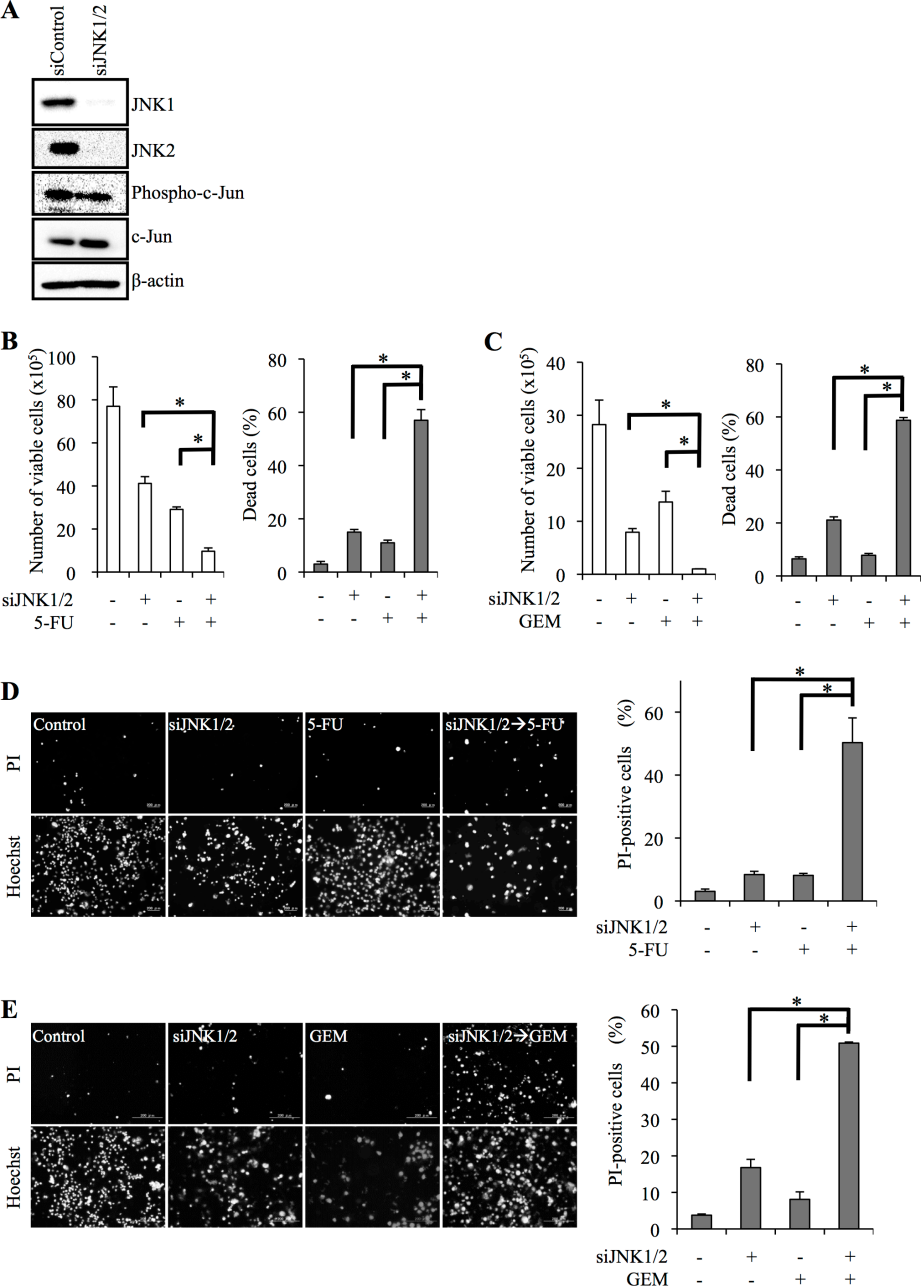
siRNA-mediated JNK knockdown sensitizes pancreatic cancer stem cells to 5-fluorouracil and gemcitabine **(A)** PANC-1 CSLCs were transiently transfected with siRNAs against JNK1 and JNK2 (siJNK1/2) or with a control siRNA (siControl), as detailed in Materials and methods. After 8 days, the transfected cells were subjected to immunoblot analyses for the expression of the indicated proteins. **(B, C)** PANC-1 CSLCs were transfected as in (A) followed by treatment with 5-fluorouracil (5-FU, 20 μM) or gemcitabine (GEM, 2 μM) as indicated for 3 days. Then, the number of viable cells (*left panels*) and the percentage of dead cells (*right panels*) were determined using trypan blue. Values represent means + SD from triplicate samples of a representative experiment repeated with similar results. *P < 0.05. **(D, E)** PANC-1 CSLCs were transfected as in (A) followed by treatment with 5-fluorouracil (5-FU, 20 μM) or gemcitabine (GEM, 2 μM) for 3 days. Then the cells treated as indicated were subjected to cell death analysis using propidium iodide (PI). *Left,* representative fluorescence images of PI-(*upper rows*) and Hoechst-(*lower rows*) positive cells are shown. *Right*, the percentage of PI-positive cells (dead cells) relative to Hoechst-positive cells (total cells) was determined. Values in the graphs represent means + SD from triplicate samples of a representative experiment repeated with similar results. *P < 0.05.

To ascertain whether the effect of SP600125 on pancreatic CSCs was mediated by inhibiting JNK activity, we next examined the effect of JNK knockdown on resistance to 5-FU/GEM. Transfection of pancreatic CSCs with combinations of siRNAs directed against *JNK1* and *JNK2* (siJNK1/2) mRNAs decreased the expression of JNK1 and JNK2 (Figure 3A; Supplementary Figure S4A), and the proportion of dead cells was substantially increased when cells were exposed to 5-FU (Figure 3B, 3D; Supplementary Figure S4B, S4D) or GEM (Figure 3C, 3E; Supplementary Figure S4C, S4E) in combination with JNK knockdown. Thus, these results strongly suggest that JNK signaling is critically involved in the chemoresistance of pancreatic CSCs to 5-FU/GEM.

### JNK contributes to the chemoresistance of pancreatic cancer stem cells through suppression of 5-fluorouracil/gemcitabine-induced increase in intracellular reactive oxygen species

**Figure 4:**
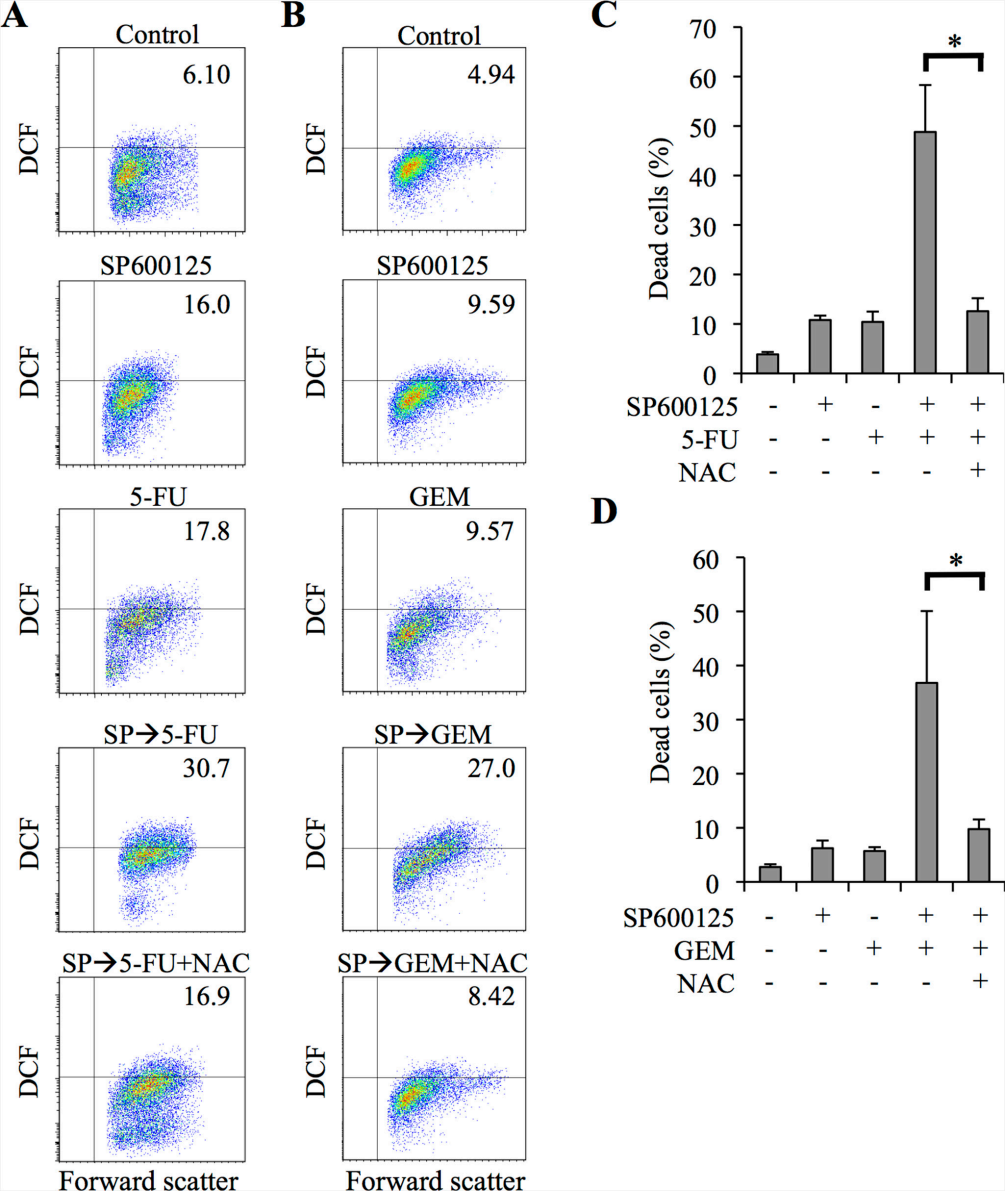
JNK inhibitor pretreatment sensitizes pancreatic cancer stem cells to 5-fluorouracil and gemcitabine in ROS - dependent manner **(A-D)** PANC-1 CSLCs pretreated with or without SP600125 (20 μM) for 3 days were, after being cultured in the presence or absence of N-acetylcysteine (NAC, 10 mM) for 30 min, further treated with or without 5-fluorouracil (5-FU, 20 μM) or gemcitabine (GEM, 2 μM) as indicated for 3 days in the absence of SP600125. Then, the cells were analyzed as follows. **(A, B)** Cells were stained with 2′,7′-dichlorofluorescein diacetate (DCF-DA) and subjected to flow cytometric analysis to detect intracellular ROS. Representative flow cytometric plots with the percentages of ROS-positive cells are shown. **(C, D)** The percentage of dead cells was determined using trypan blue. Values represent means + SD from triplicate samples of a representative experiment repeated with similar results. *P < 0.05.

We next investigated the mechanism by which JNK contributes to the chemoresistance of pancreatic CSCs. Since reactive oxygen species (ROS) have been implicated in the cytotoxicity of chemotherapeutic agents such as 5-FU and GEM [16–21], we examined the intracellular ROS levels of pancreatic CSCs after drug treatment. We noted that treatment of PANC-1 CSLCs with 5-FU (Figure 4A) or GEM (Figure 4B) alone modestly increased the proportion of cells with increased intracellular ROS compared with controls. We also noted at the same time that SP600125 treatment of PANC-1 CSLCs increased the intracellular ROS levels (Figure 4A, 4B). Strikingly, pretreating of PANC-1 CSLCs with SP600125 combined with 5-FU/GEM treatment remarkably increased the proportion of ROS-positive cells compared with 5-FU/GEM treatment alone (Figure 4A, 4B).

To determine whether the increase in intracellular ROS caused by SP600125 pretreatment has a role in SP600125-mediated sensitization of PANC-1 CSLCs to 5-FU and GEM, we next examined the effect of the antioxidant N-acetylcysteine (NAC), which scavenges free radicals by increasing intracellular glutathione levels (GSH) [22]. NAC, which effectively suppressed the increase in intracellular ROS, inhibited sensitization of pancreatic CSCs to 5-FU and GEM by SP600125; NAC treatment significantly decreased the proportion of dead cells after treatment of PANC-1 CSLCs with 5-FU (Figure 4C; Supplementary Figure S5) and GEM (Figure 4D) in combination with SP600125. Essentially similar results were obtained when PSN-1 CSLCs (Supplementary Figure S6A – S6D) were used instead of PANC-1 CSLCs. Together, the results suggested that SP600125 sensitized pancreatic CSCs to 5-FU and GEM through promotion of 5-FU/GEM-induced increase in intracellular ROS.

**Figure 5:**
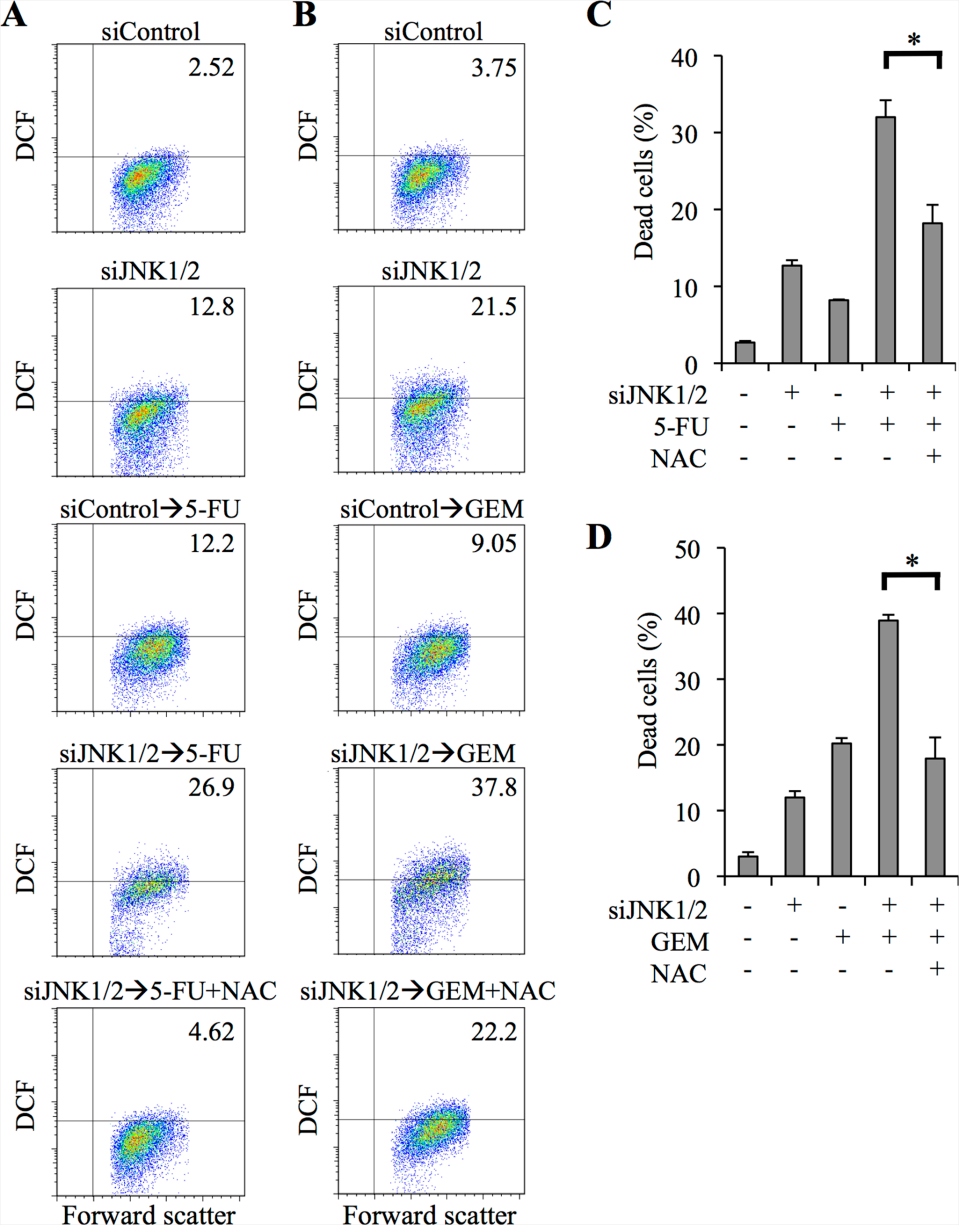
siRNA-mediated JNK knockdown sensitizes pancreatic cancer stem cells to 5-fluorouracil and gemcitabine in ROS - dependent manner **(A-D)** PANC-1 CSLCs were transiently transfected with siRNAs against JNK1 and JNK2 (siJNK1/2) or with a control siRNA (siControl), as detailed in Materials and methods. After 8 days, the transfected cells were cultured in the presence or absence of N-acetylcysteine (NAC, 10 mM) for 30 min and further treated with or without 5-fluorouracil (5-FU, 20 μM) or gemcitabine (GEM, 2 μM) as indicated for 3 days. Then, the cells were analyzed as follows. **(A, B)** Cells were stained with 2′,7′-dichlorofluorescein diacetate (DCF-DA) and subjected to flow cytometric analysis to detect intracellular ROS. Representative flow cytometric plots with the percentages of ROS-positive cells are shown. **(C, D)** The percentage of dead cells was determined using trypan blue. Values represent means + SD from triplicate samples of a representative experiment repeated with similar results. *P < 0.05.

To ascertain that the effect of SP600125 on ROS accumulation induced by 5-FU/GEM, as well as on ROS-mediated sensitization of pancreatic CSCs to 5-FU/GEM, was dependent on the inhibition of JNK, we next examined the effect of JNK knockdown on the intracellular ROS level of PANC-1 CSLCs treated with 5-FU or GEM. Transient transfection of PANC-1 CSLCs with siJNK1/2 increased the proportion of ROS-positive cells following treatment with 5-FU/GEM compared with 5-FU/GEM treatment combined with control siRNA transfection (Figure 5A, 5B). Importantly, we confirmed that NAC treatment inhibited the augmentation of 5-FU/GEM-induced increase in intracellular ROS and cytotoxicity by JNK knockdown (Figure 5C, 5D). Together, these results suggested that JNK may contribute to the chemoresistance of pancreatic CSCs by preventing drug-induced increase in intracellular ROS.

### JNK inhibition followed by treatment with 5-fluorouracil or gemcitabine in the absence of simultaneous JNK inhibition does not cause toxicity to normal human fibroblasts

**Figure 6:**
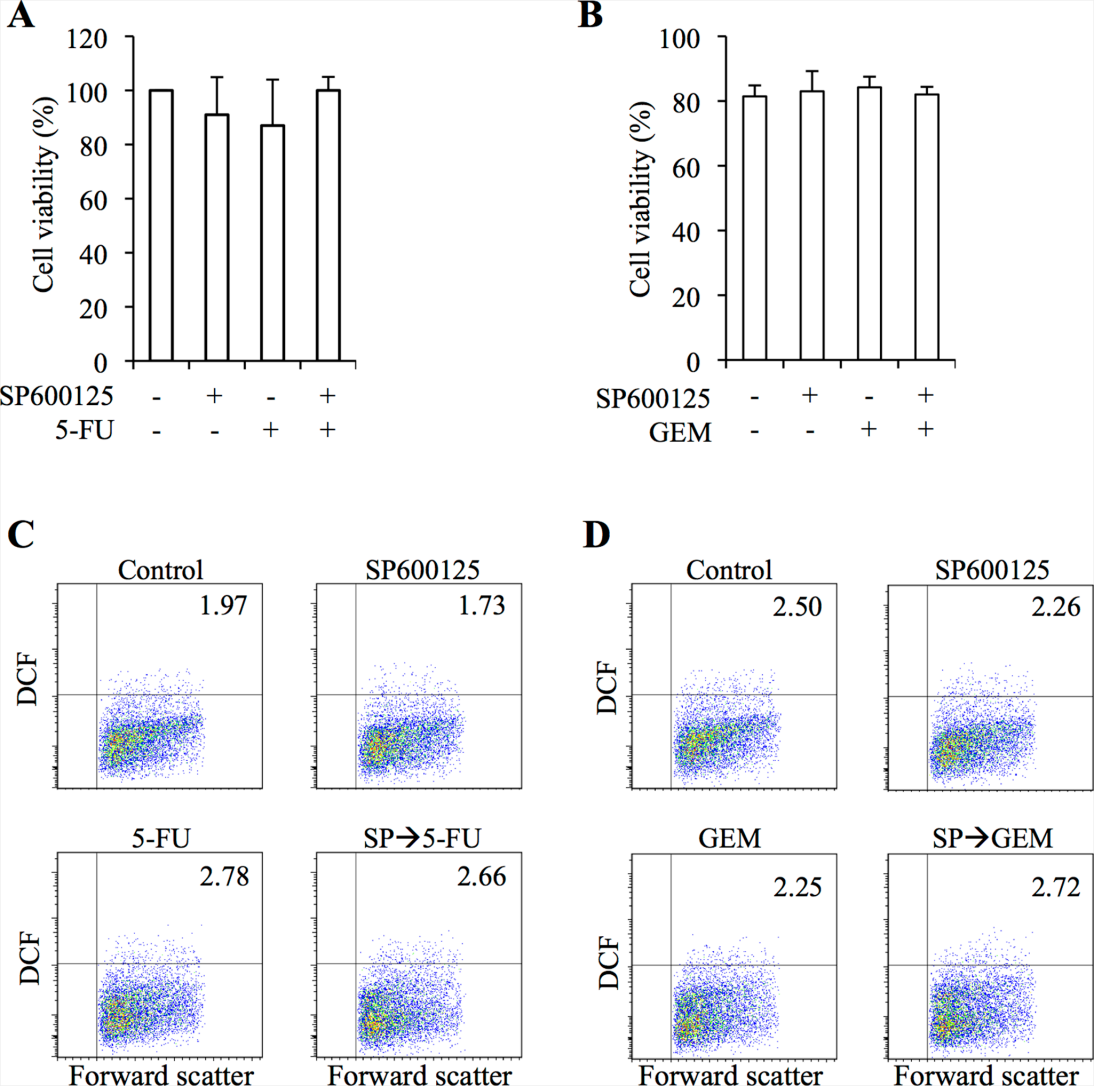
JNK inhibitor pretreatment does not increase the toxic effects of 5-fluorouracil and gemcitabine on normal human fibroblasts **(A-D)** IMR90 normal human fibroblasts were pretreated with or without SP600125 (SP, 20 μM) for 3 days and subsequently treated with or without 5-fluorouracil (5-FU, 20 μM) or gemcitabine (GEM, 2 μM) as indicated in the absence of SP600125 for 3 days. The cells were then analyzed as follows. **(A, B)** Cell viability was determined using trypan blue. Values represent means + SD from triplicate samples of a representative experiment repeated with similar results. **(C, D)** Cells were stained with 2′,7′-dichlorofluorescein diacetate (DCF-DA) and subjected to flow cytometric analysis to detect intracellular ROS. Representative flow cytometric plots with the percentages of ROS-positive cells are shown.

We next evaluated the effects of combination treatment with SP600125 and 5-FU/GEM on IMR90 normal human lung fibroblasts. When IMR90 cells were treated with the drugs at their respective highest concentrations used in combination treatments in this study (20 μM for SP600125, 20 μM for 5-FU, and 2 μM for GEM), the viability of IMR90 cells was not appreciably affected either by single or combination treatments, so long as SP600125 treatment was applied prior to 5-FU or GEM in case of combination treatments (Figure 6A, 6B). Of note, the intracellular ROS level of IMR90 cells remained unchanged irrespective of whether the drugs were applied alone or in combination, which may explain the lack of toxicity of these drugs to IMR90 cells (Figure 6C, 6D). We also asked here whether the timing of SP600125 treatment affects its toxicity to IMR90 cells. Whereas “pre-treatment” and “pre- and co-treatment” of SP600125 were similarly effective at sensitizing pancreatic CSCs to 5-FU (Supplementary Figure S3), “pre- and co-treatment” but not “pre-treatment” turned out to have a growth inhibitory effect on IMR90 cells though neither treatment induced cell death (Supplementary Figure S7A, S7B). Thus, the results suggest that the “pre-treatment” protocol might have the widest therapeutic window when JNK inhibition is to be combined with 5-FU/GEM to target pancreatic CSCs.

## DISCUSSION

Pancreatic cancer is one of the most malignant human cancers, which is so often unresectable already at the time of diagnosis and, above all, responds poorly to either chemotherapy or radiotherapy [3–5]. A growing body of evidence has come to suggest that pancreatic CSCs, endowed with both tumor-initiating capacity as well as with high therapy resistance, is among the key factors underlying the extreme refractoriness of pancreatic cancer to conventional cancer therapies. Identification of the molecules and/or signaling pathways that confer chemoresistance on pancreatic CSCs, therefore, is expected to contribute to better management of pancreatic cancer. However, currently, the molecular basis of the increased chemoresistance of pancreatic CSCs remains poorly understood.

Here in this study, we first found that pancreatic CSCs, which we have shown to have activated JNK [15], were highly resistant to 5-FU and GEM compared with their non-stem counterparts, essentially consistent with earlier reports [13, 14]. We then demonstrated that inhibition of the activated JNK in pancreatic CSCs sensitized them to 5-FU and GEM and that JNK inhibition did so through elevation of the intracellular ROS level in combination with these chemotherapeutic agents. These results suggest that a JNK-regulated ROS defense mechanism plays a key role in the chemoresistance of pancreatic CSCs and that targeting this mechanism may be a useful measure to overcome the chemoresistance of pancreatic CSCs.

Although JNK was originally identified as a positive regulator of cell death and overwhelming evidence does support the idea, JNK has also been shown to have a pro-survival function depending on the context [23–25]. Indeed, there are studies suggesting that the JNK pathway may play a positive role in chemoresistance of some cancer types [26]. However, the role of JNK in chemoresistance/sensitivity of pancreatic cancer cells, let alone pancreatic CSCs, to clinically relevant chemotherapeutic agents has remained largely unknown except for a report showing that GEM-induced apoptosis of pancreatic cancer cells was dependent on JNK-mediated DUSP1 expression [27]. In this regard, the present study is the very first to demonstrate that JNK has a protective role against 5-FU or GEM in pancreatic cancer cells, more specifically, in pancreatic CSCs. On the other hand, although the mechanisms involved in pancreatic CSC chemoresistance are only beginning to be understood, recent studies have suggested that uPA and c-Met may also be involved in the chemoresistance of pancreatic CSCs, which is quite intriguing in light of the previous observations that JNK could function upstream and downstream of uPA and c-Met, respectively [28–30]. Although not pursued in the present study, investigation on the possible functional links among uPA, c-Met, and JNK may help better understand the whole picture of the molecular mechanism underlying the chemoresistance of pancreatic CSCs.

In this study, we have provided evidence that JNK contributes to the chemoresistance of pancreatic CSCs through suppression of the intracellular ROS level. It has been demonstrated that CSCs have lower intracellular ROS levels and that the low ROS levels are associated with the radioresistance of CSCs [31, 32]. In contrast to radioresistance, however, the role of enhanced ROS defense of CSCs in chemoresistance has yet to be established. Significantly, lines of evidence suggest that ROS may participate in the cytotoxic mechanisms of chemotherapeutic agents such as 5-FU and GEM [17, 33–37], lending support to the idea that cellular antioxidant capacity may determine cells' sensitivity/resistance to such chemotherapeutic agents. The results of the present study, indicating that the increase in intracellular ROS caused by JNK inhibition sensitizes pancreatic CSCs to 5-FU and GEM, are quite in line with the idea. Although only pancreatic CSCs were examined in the present study, future studies may reveal that this paradigm can be extended to CSCs of other cancer types. It would also be worth mentioning here that our results clearly demonstrated the key role of JNK in maintaining the intracellular ROS level low in pancreatic CSCs. In sharp contrast to the well-documented role of ROS in JNK activation [38, 39], the role of JNK in the control of the intracellular ROS level remains largely uninvestigated. Yet there have been two studies reporting the ROS-inhibitory role of JNK. Interestingly, these two studies showed that activated JNK was localized to mitochondria and that JNK inhibition led to accumulation of mitochondrial superoxide [40, 41], pointing to the intriguing possibility that JNK may have a role in keeping the mitochondrial ROS level in check.

Unlike in normal pancreas, JNK is activated in the majority of pancreatic cancers [42]. The significance of JNK activation in pancreatic cancer biology remained unclear, but we have only recently demonstrated that JNK activation by KRas, which is mutationally activated in the majority of pancreatic cancers, plays a key role in the maintenance of self-renewal and tumor-initiating capacity of pancreatic CSCs [15]. The findings of the present study now add a new role of JNK in pancreatic CSCs, namely, the maintenance of chemoresistance. The role of KRas in the chemoresistance of pancreatic CSCs still remains to be explored, but a previous study showed that Ras inhibition using farnesylthiosalicylic acid sensitized pancreatic cancer cells to GEM [43]. It might therefore be tempting to speculate that KRas contributes to the chemoresistance of pancreatic CSCs as well through the activation of the JNK-ROS defense axis.

Of importance from a therapeutic perspective, we found in this study that JNK inhibitor pre-treatment as well as pre- and co-treatment was superior to co-treatment in augmenting the cytotoxicity of 5-FU to pancreatic CSCs, whereas pre- and co-treatment was apparently more toxic to normal human fibroblasts than the others. The results, though being in vitro findings, suggest that a time-staggered treatment protocol might be beneficial in maximizing the therapeutic effect while minimizing adverse effects if JNK inhibition is to be combined with FU-based regimens (and possibly GEM-based regimens) in future pancreatic cancer treatment. As an explanation for the superior effect of JNK inhibitor pre-treatment, we found that the increase in the intracellular ROS level caused by JNK inhibition persisted at least for days after cessation of JNK inhibition (S.S, and M.O., unpublished observation), suggesting that “rewiring” of signaling pathways [44] may have taken place with some stability during the pre-treatment period, i.e., by the time cells were treated with 5-FU. On the other hand, GEM for instance reportedly exerts its cytotoxic effect on lung cancer cells through activation of JNK [45], suggesting the possibility that co-treatment could render the cells even more resistant to chemotherapeutic agents in case their cytotoxic effect is JNK-dependent. If we take this possibility into consideration, the pre-treatment protocol may be superior to the others in sensitizing pancreatic CSCs to chemotherapeutic agents, which is to be investigated using GEM in combination with JNK inhibitor treatment.

In conclusion, we have elucidated in this study the mechanism underlying the increased chemoresistance of pancreatic CSCs, namely, JNK-mediated suppression of the intracellular ROS level. Although preclinical studies are warranted, our findings also suggest that targeting JNK in combination with FU- or GEM-based regimens may be a promising approach to elimination of pancreatic CSCs and therefore successful treatment of pancreatic cancer.

## MATERIALS AND METHODS

### Antibodies and reagents

Anti-c-Jun (#9165) and anti-phospho-c-Jun (#9261) antibodies were purchased from Cell Signaling Technology, Inc. (Beverly, MA, USA). Anti-β-actin (A1978) was from Sigma (St. Luis, MO, USA). Anti-JNK1 (sc-474) and anti-JNK2 (sc-7345) were from Santa Cruz Biotechnology, Inc. (Santa Cruz, CA, USA). SP600125 was purchased from Calbiochem (La Jolla, CA, USA) and was dissolved in dimethylsulfoxide (DMSO) to prepare a 50 mM stock solution. 5-FU, GEM, 2′,7′-dichlorofluorescein diacetate (DCF-DA), and N-acetyl-L-cysteine (NAC) were from Sigma and were dissolved in DMSO to prepare 200 mM, 1 mM, 20 mM, and 5 M stock solutions, respectively.

### Cell culture

The establishment of PANC-1 CSLCs and PSN-1 CSLCs, CSCs derived from human pancreatic cancer cell lines PANC-1 and PSN-1, respectively, was previously reported [15]. The authenticity of PANC-1 CSLCs and PSN-1 CSLCs as cells derived from PANC-1 and PSN-1, respectively, was verified by genotyping of short tandem repeat (STR) loci (Bio-Synthesis, Inc., Lewisville, TX, USA) followed by comparison to the ATCC STR database for Human Cell Lines. Unless otherwise indicated, these pancreatic CSCs were stably maintained and used for experiments under the monolayer stem cell culture condition, as previously described [15]. Briefly, cells were cultured on collagen-I-coated dishes (IWAKI, Tokyo, Japan) in the stem cell culture medium (DMEM/F12 medium supplemented with 1% B27 [Gibco-BRL, Carlsbad, CA, USA], 20 ng/mL EGF and FGF2 [Peprotech, Inc., Rocky Hill, NJ, USA], D-(+)-glucose [final concentration, 26.2 mM], L-glutamine [final concentration, 4.5 mM], 100 units/mL penicillin and 100 μg/mL streptomycin). In principle, the stem cell culture medium was changed every 3 days, and EGF and FGF2 were added to the culture medium every day. Differentiation of pancreatic CSCs was induced by culturing the cells under the differentiation-inducing condition (DMEM/F12 containing 10% FBS) for 2 weeks, as previously described [15]. Normal human IMR90 fetal lung fibroblasts [46, 47] were obtained from American Type Culture Collection and maintained in DMEM/F12 supplemented with 10% fetal bovine serum (FBS) and 100 units/mL penicillin and 100 μg/mL streptomycin. All IMR90 experiments were performed using low passage number (less than 9) cells.

### Gene silencing by siRNA

siRNAs against human *JNK1* (VHS40722) and *JNK2* (VHS40726) as well as Medium GC Duplex #2 of Stealth RNAi™ siRNA Negative Control Duplexes (as a control for siRNA experiments) were purchased from Invitrogen Life Technologies (Carlsbad, CA, USA). Transfection of siRNAs was performed using Lipofectamine RNAiMAX™ (Life Technologies) according to the manufacturer's instructions. To achieve sustained knockdown of the target genes, siRNA transfection was repeated 4 days after the initial transfection.

### 5-Fluorouracil and gemcitabine treatment and cell viability assays

Unless otherwise indicated, throughout this study, PANC-1 CSLCs were treated with 20 μM of 5-FU or 2 μM of GEM for 3 days. PSN-1 CSLCs were treated with 2.5 μM of 5-FU or 0.25 μM of GEM for 3 days. In each set of treatment, cells were examined for their viability at the end of pretreatment (i.e., SP600125 treatment [20 μM for PANC-1 CSLCs, 10 μM for PSN-1 CSLCs] and/or knockdown of JNK1/2), and equal numbers of viable cells were used for subsequent 5-FU, GEM, or control (DMSO) treatment. Viable and dead cells were identified by their ability and inability to exclude vital dyes, respectively [48]. In brief, cells were stained with 0.2% trypan blue, and the numbers of viable and dead cells were determined using a hemocytometer. Cell viability (%) was defined as 100 × ‘the number of viable cells’/ (‘the number of viable cells’ + ‘the number of dead cells’), whereas the percentage of dead cells was defined as 100 × ‘the number of dead cells’/(‘the number of viable cells’ + ‘the number of dead cells’). Alternatively, cells were incubated in situ with propidium iodide (PI, 1 μg/ml) and Hoechst 33342 (10 μg/ml) for 10 min at 37°C in the CO_2_ incubator, to stain dead cells and the cell nuclei, respectively. Then the numbers of PI- and Hoechst-positive cells were scored under a fluorescence microscope (CKX41; Olympus, Tokyo, Japan), and the percentage of PI-positive cells (dead cells) against Hoechst-positive cells (total cells) was determined.

### Detection and measurement of intracellular ROS

Cells were incubated in the stem cell culture medium containing 10 μM DCF-DA for 10 min at 37°C, washed twice with phosphate-buffered saline (PBS), and re-suspended in PBS. The cells were then subjected to flow cytometric analysis, during which care was taken not to expose the samples to light. For flow cytometric analysis, at least 1 × 10^4^ cells were evaluated and gated using side and forward scatters to identify viable cell populations. All flow cytometric experiments were run on FACSCanto™ II Flow Cytometer (BD Biosciences, Franklin Lakes, NJ, USA) and the data were analyzed using FlowJo software, version7.6.5 (Treestar Inc., Ashland, OR, USA).

### Immunoblot analysis

Cells were washed with ice-cold PBS and lysed in RIPA buffer (10 mM Tris-HCl [pH 7.4], 0.1% SDS, 0.1% sodium deoxycholate, 1% NP-40, 150 mM NaCl, 1 mM EDTA, 1.5 mM Na_3_VO_4_, 10 mM NaF, 10 mM sodium pyrophosphate, 10 mM sodium β-glycerophosphate and 1% protease inhibitor cocktail set III [Calbiochem]). After centrifugation for 10 min at 14,000 × g at 4°C, the supernatants were recovered as the cell lysates, and the protein concentration of the cell lysates was determined by the BCA protein assay kit (Pierce Biotechnology, Inc., Rockford, IL, USA). Cell lysates containing equal amounts of protein were separated by SDS-PAGE and transferred to a polyvinylidene difluoride membrane. The membrane was probed with a primary antibody and then with an appropriate HRP-conjugated secondary antibody according to the protocol recommended by the manufacturer of each antibody. Immunoreactive bands were visualized using Immobilon Western Chemiluminescent HRP Substrate (Millipore, Billerica, MA, USA).

### Statistical analysis

Results are expressed as means and standard deviation (SD), and differences were compared using the 2-tailed Student's *t*-test. *P*-values < 0.05 were considered statistically significant and indicated with asterisks in the figures.

## SUPPLEMENTARY FIGURES


