# Supplementary material for: JNK suppression of chemotherapeutic agents-induced ROS confers chemoresistance on pancreatic cancer stem cells
Source: Oncotarget. 2014 Nov 19;6(1):458–70. doi: 10.18632/oncotarget.2693 (PMC4381607; doi:10.18632/oncotarget.2693)
Supplement: Supplementary file 1 [file oncotarget-06-458-s001.pdf]

SUPPLEMENTARY FIGURES

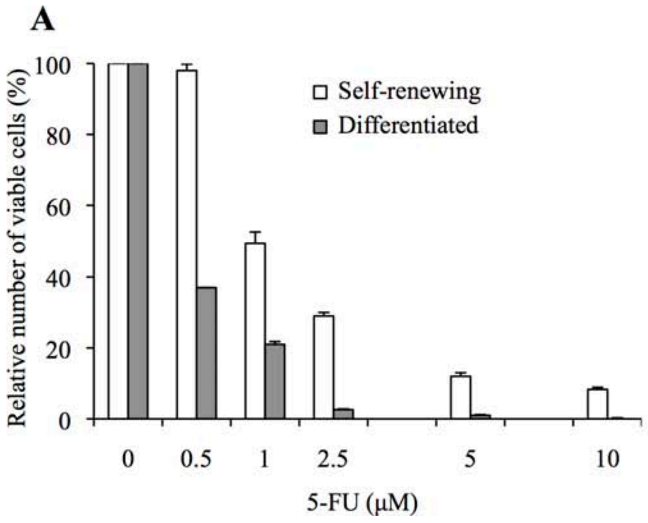

Supplementary Figure S1: PSN-1 CSLCs show higher resistance to 5-fluorouracil and gemcitabine than their non-stem cell counterparts. **(A, B)** PSN-1 CSLCs, either maintained under the stem cell culture condition (Self-renewing, white bars) or induced to undergo differentiation in the presence of serum (Differentiated, gray bars), were treated with the indicated concentrations of 5-fluorouracil (A, 5-FU) or gemcitabine (B, GEM) for 3 days. Then, the number of viable cells was determined using trypan blue as a vital dye. Values in the graphs are expressed as relative to controls (i.e., drug concentration = 0 μM) and represent means + SD from triplicate samples of a representative experiment repeated with similar results.

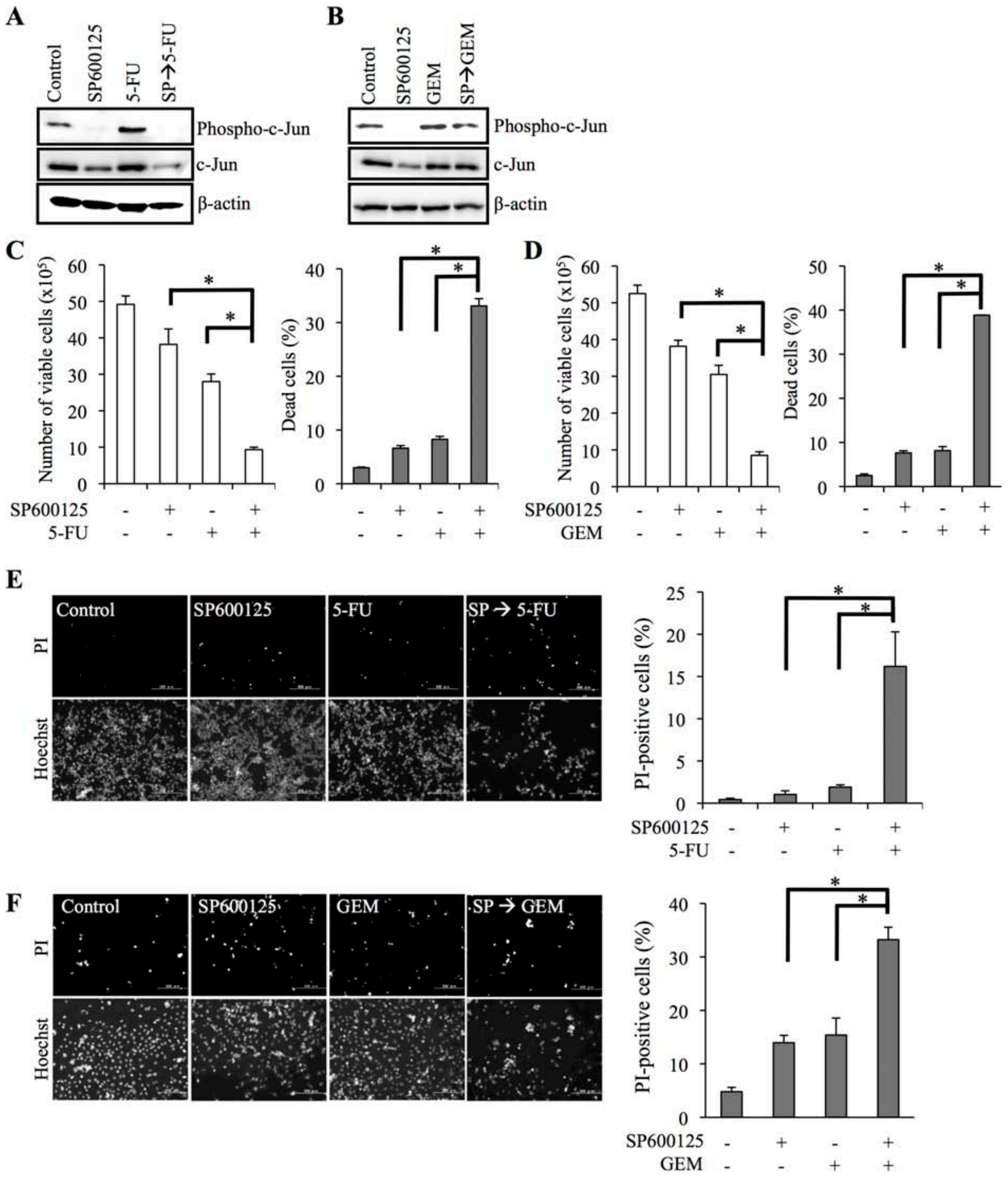

Supplementary Figure S2: JNK inhibitor pretreatment sensitizes PSN-1 CSLCs to 5-fluorouracil and gemcitabine. **(A-F)** PSN-1 CSLCs pretreated with or without SP600125 (SP, 10 µM) for 3 days and subsequently treated with or without 5-fluorouracil (5-FU, 2.5 µM) or gemcitabine (GEM, 0.25 µM) as indicated for 3 days in the absence of SP600125 were analyzed as follows. **(A, B)** Cells were subjected to immunoblot analyses for the expression of phospho- and total c-Jun. **(C, D)** The number of viable cells (*left panels*) and the percentage of dead cells (*right panels*) were determined using trypan blue as a vital dye. Values represent means + SD from triplicate samples of a representative experiment repeated with similar results. *$P < 0.05$. **(E, F)** Cells were subjected to cell death analysis using propidium iodide (PI) as a vital dye. *Left,* representative fluorescence images of PI- (*upper rows*) and Hoechst- (*lower rows*) positive cells are shown. *Right*, the percentage of PI-positive cells (dead cells) relative to Hoechst-positive cells (total cells) was determined. Values in the graphs represent means + SD from triplicate samples of a representative experiment repeated with similar results. *$P < 0.05$.

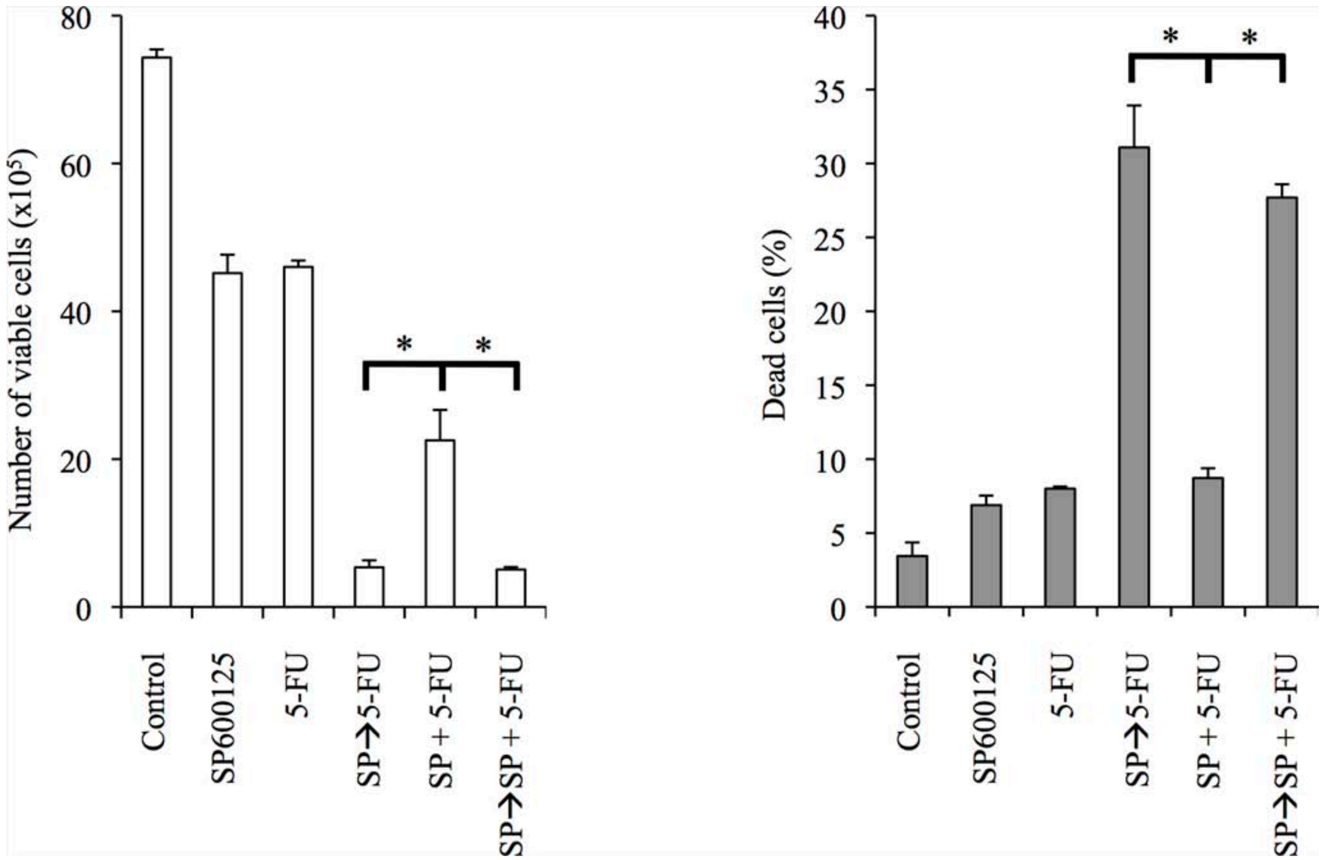

Supplementary Figure S3: Effective sensitization of PANC-1 CSLCs to 5-fluorouracil by SP600125 is dependent on the timing of its treatment. PANC-1 CSLCs pretreated with or without SP600125 (SP, 20 μM) for 3 days were subsequently treated with or without 5-FU (20 μM) in the presence or absence of SP600125. The number of viable cells (*left*) and the percentage of dead cells (*right*) were determined using trypan blue. Values represent means + SD from triplicate samples of a representative experiment repeated with similar results. *$P < 0.05$.

Note that;
SP→5-FU indicates that cells were pretreated with SP600125 for 3 days and then with 5-FU in the absence of SP600125 for another 3 days.
SP+5-FU indicates that cells were simultaneously treated with SP600125 and 5-FU for 3 days.
SP5→SP+5-FU indicates that cells were pretreated with SP600125 for 3 days and then with 5-FU in the presence of SP600125 for another 3 days.

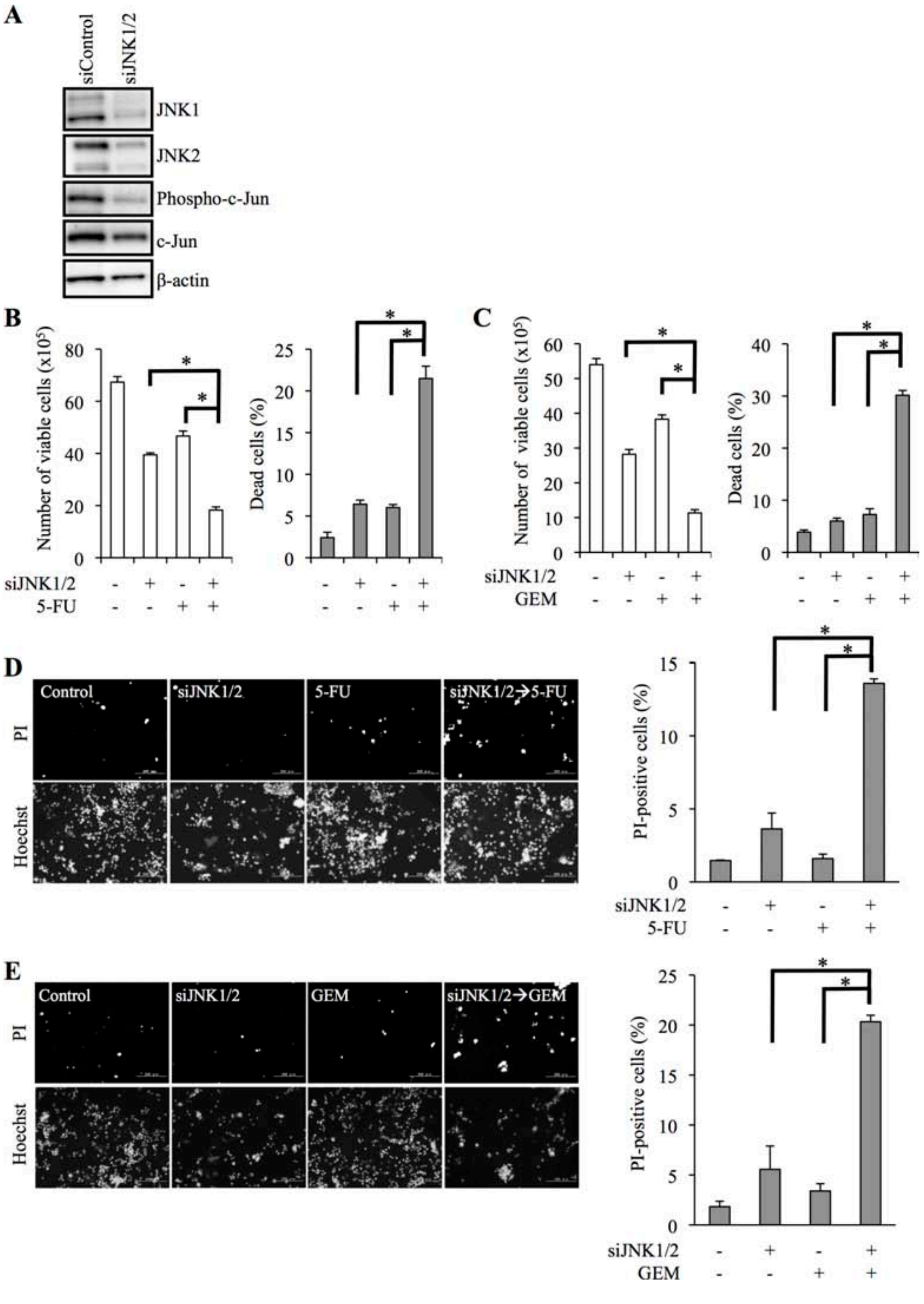

Supplementary Figure S4: siRNA-mediated JNK knockdown sensitizes PSN-1 CSLCs to 5-fluorouracil and gemcitabine. (A) PSN-1 CSLCs were transiently transfected with siRNAs against JNK1 and JNK2 (siJNK1/2) or with a control siRNA (siControl), as detailed in Materials and methods. After 8 days, the transfected cells were subjected to immunoblot analyses for the expression of the indicated proteins. **(B, C)** PSN-1 CSLCs were transfected as in (A) and then treated with 5-fluorouracil (5-FU, 2.5 μM) or gemcitabine (GEM, 0.25 μM) as indicated for 3 days. Then, the number of viable cells (*left panels*) and the percentage of dead cells (*right panels*) were determined using trypan blue. Values represent means + SD from triplicate samples of a representative experiment repeated with similar results. *$P < 0.05$. **(D, E)** PSN-1 CSLCs were transfected as in (A) and then treated with 5-fluorouracil (5-FU, 2.5 μM) or gemcitabine (GEM, 0.25 μM) as indicated for 3 days. Then the cells were subjected to cell death analysis using propidium iodide (PI). *Left,* representative fluorescence images of PI- (upper rows) and Hoechst- (lower rows) positive cells are shown. *Right*, the percentage of PI-positive cells (dead cells) relative to Hoechst-positive cells (total cells) was determined. Values in the graphs represent means + SD from triplicate samples of a representative experiment repeated with similar results. *$P < 0.05$.

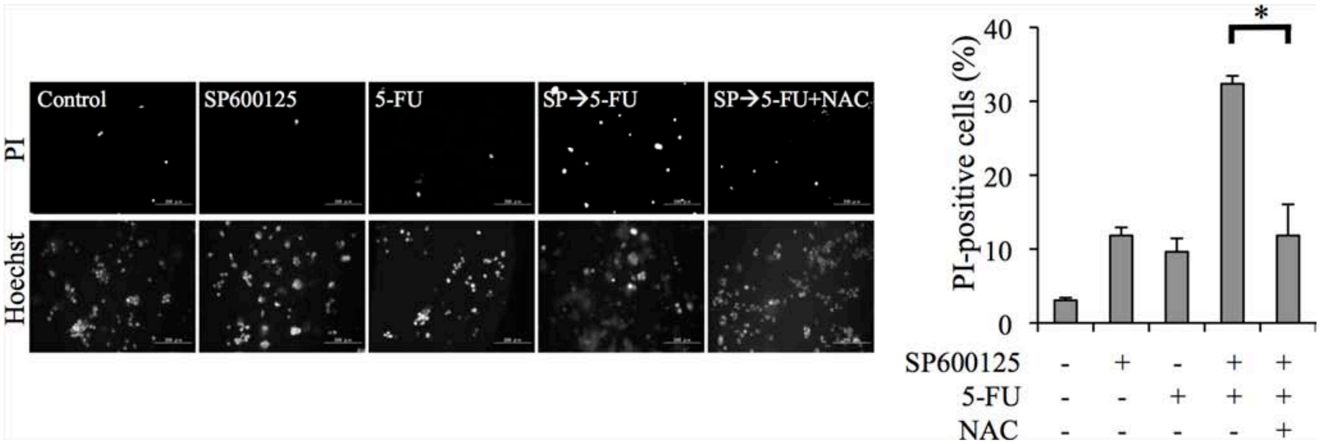

Supplementary Figure S5: JNK inhibitor pretreatment sensitizes pancreatic cancer stem cells to 5-fluorouracil in ROS - dependent manner. PANC-1 CSLCs pretreated with or without SP600125 (20 μM) for 3 days were, after being cultured in the presence or absence of N-acetylcysteine (NAC, 10 mM) for 30 min, further treated with or without 5-fluorouracil (20 μM) for 3 days in the absence of SP600125. The 5-fluorouracil (5-FU)-treated cells were subjected to cell death analysis using propidium iodide (PI). *Left,* representative fluorescence images of PI- (upper rows) and Hoechst- (lower rows) positive cells are shown. *Right*, the percentage of PI-positive cells (dead cells) relative to Hoechst-positive cells (total cells) was determined. Values in the graph represent means + SD from triplicate samples of an experiment. *$P < 0.05$.

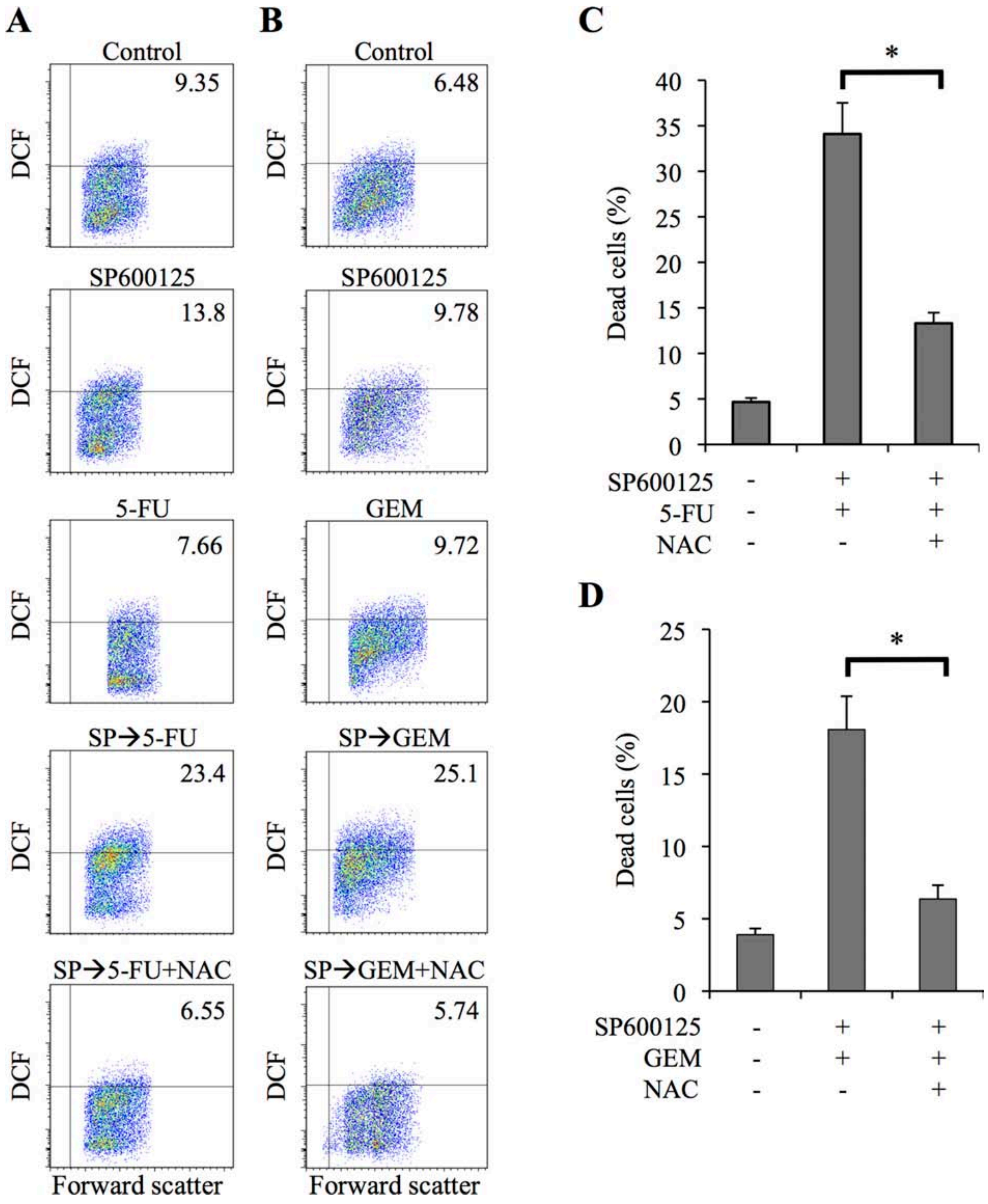

Supplementary Figure S6: JNK inhibitor pretreatment sensitizes PSN-1 CSLCs to 5-fluorouracil and gemcitabine in ROS - dependent manner. **(A-D)** PSN-1 CSLCs pretreated with or without SP600125 (10 μM) for 3 days were, after being cultured in the presence or absence of N-acetylcysteine (NAC, 10 mM) for 30 min, further treated with or without 5-fluorouracil (5-FU, 2.5 μM) or gemcitabine (GEM, 0.25 μM) as indicated for 3 days in the absence of SP600125. Then, the cells were analyzed as follows. **(A, B)** Cells were stained with 2′,7′-dichlorofluorescein diacetate (DCF-DA) and subjected to flow cytometric analysis to detect intracellular ROS. Representative flow cytometric plots with the percentages of ROS-positive cells are shown. **(C, D)** The percentage of dead cells was determined using trypan blue. Values represent means + SD from triplicate samples of an experiment. *$P < 0.05$.

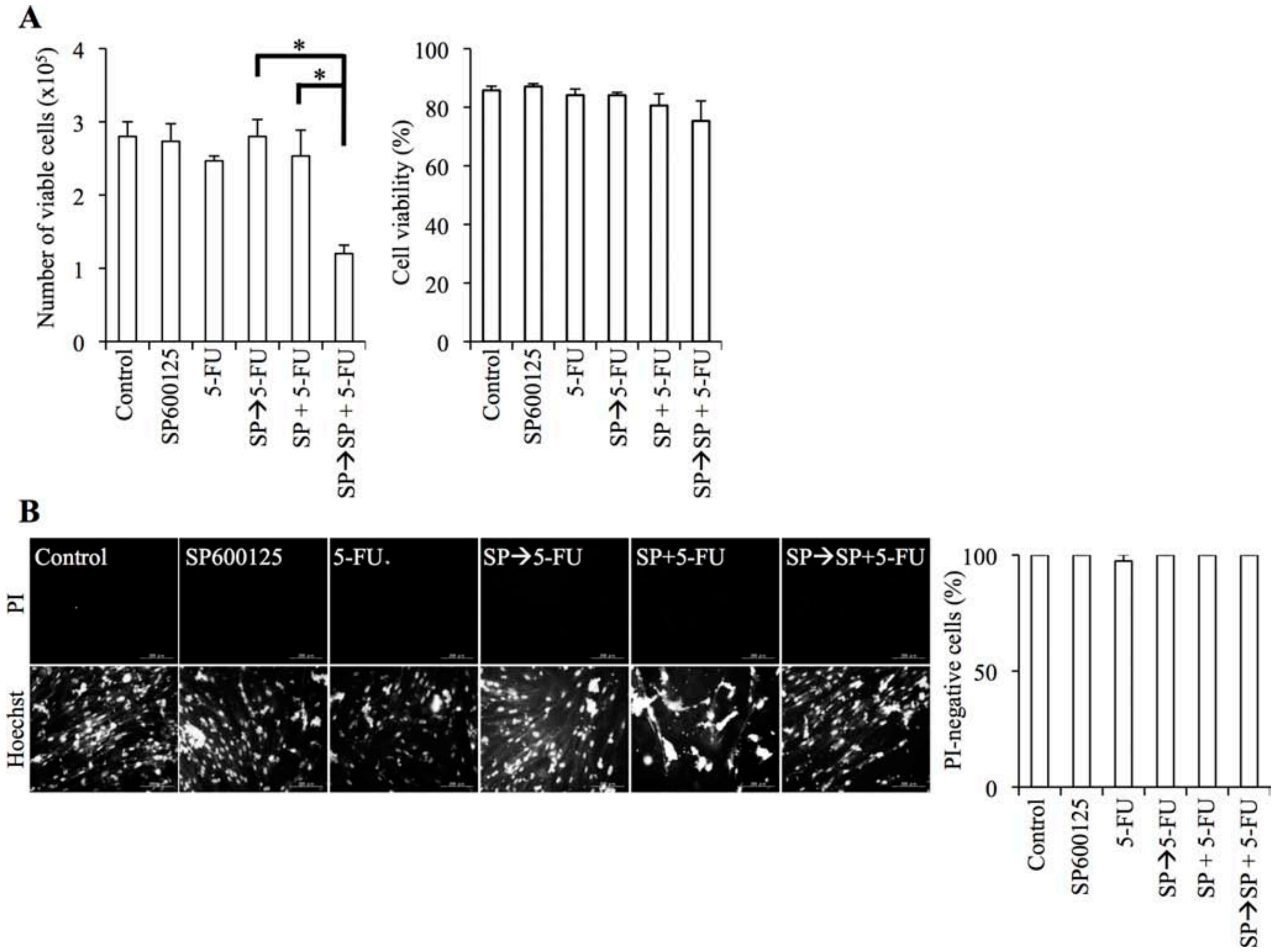

Supplementary Figure S7: The growth inhibitory effect of combinatorial treatment with SP600125 and 5-fluorouracil is dependent on the timing of treatment. (A) IMR90 normal human fibroblasts pretreated with or without SP600125 (SP, 20 μM) for 3 days were subsequently treated with or without 5-FU (20 μM) in the presence or absence of SP600125. The number of viable cells (*left*) and cell viability (*right*) were determined using trypan blue. Values represent means + SD from triplicate samples of a representative experiment repeated with similar results. **P* < 0.05. **(B)** Cells treated as in (A) were subjected to cell death analysis using propidium iodide (PI). *Left,* representative fluorescence images of PI- (upper rows) and Hoechst- (lower rows) positive cells are shown. *Right*, the percentage of PI-negative cells (viable cells) relative to Hoechst-positive cells (total cells) was determined. Values in the graph represent means + SD from triplicate samples of a representative experiment repeated with similar results. **P* < 0.05.
